# Cæsarean Section1Presented at the Fourteenth Annual Meeting of the Iowa State Veterinary Medical Association, Des Moines, Iowa, February 11 and 12, 1902.

**Published:** 1902-05

**Authors:** H. L. Stewart

**Affiliations:** Lacona, Iowa


### CÆSAREAN SECTION.[1]

By H. L. Stewart, M.D.C.,
LACONA, IOWA.

Cæsarean section, or gastrohysterectomy, is an operation which has for its object the removal of the fœtus or fœtuses from the uterus of the parent when they cannot be removed in the natural way, and consists of making an opening through the abdominal wall and into the uterus for the purpose of such removal. This is quite a serious operation. It has been resorted to from a very early period in the human family, even in the days of Apollo and Julius Cæsar. Since these early times it has often been practised upon women. When it was first attempted in veterinary practice is not exactly known, but it is thought to have been practised by the Greek veterinarians at a very early date. Nothing definite is known of it until within the last century and a half, and it is not known to have been performed on the living animal until within less than a century. The operation is not frequently necessary on the mare and cow, and is less frequently successful on these animals; but on the smaller animals, especially swine, it is more successful. This is thought to be due to the attachment of the placenta to the uterus, which renders the animal less liable to septic infection through injury to the uterus. It is an operation which should be resorted to only in cases where the fœtus is alive and delivery by the natural passage is impossible or so difficult or dangerous that the mother incurs as much risk as she would from the operation; or where the owner prefers to save the offspring alive rather than incur the risk of losing both, the progeny being the more valuable; or when there are fractures or exostoses of the pelvis which greatly diminish the canal; or where there is protrusion of a large quantity of the bowels, as in one case which I had, and in which the foal was promptly removed and saved, although no effort was made to save the mare, or in extra-uterine pregnancy or torsion of the uterus. The operation is also indicated where the animal is near the termination of pregnancy and is too sick to live, or injured so that recovery is impossible, or if the animal has just died or is dying. The foal soon perishes when it cannot be born, but the calf may live for some time, varying from three minutes to a much longer time after the death of the mother. Puppies have been

[1] Presented at the Fourteenth Annual Meeting of the Iowa State Veterinary Medical Association, Des Moines, Iowa, February 11 and 12, 1902.

taken from the uterus alive eight or ten minutes after the death of the mother. Few cases are on record of successful gastrohysterectomy on cows and mares; but with the pig it is quite different. While the pig seems to be of little significance, and an animal that the veterinary practitioner is seldom called to see, yet in these days of high values, when a hog is often worth from $20 to $30 or more, it is deserving of our attention.

In all cases where the operation is decided upon no time should be lost in practising it if we wish to preserve the progeny and the mother. A clean and comfortable place should be selected. The animal should be placed on its right side and the left posterior limb fastened to something sufficient to hold it about level with the uppermost side of the patient. The hair should be clipped off over considerable space in the flank, or, better, clip the hair off before the animal is cast, if it can be done. The side should be well disinfected at the seat of the operation. A solution of creolin is perhaps the best. From the beginning use all measures to prevent septic metritis, peritonitis, or nervous exhaustion. With a scalpel make a liberal incision in the flank, as for ovariotomy, but make it longer. Twist all bleeding vessels, and then open the peritoneum; then, with the hand, reach in through the opening which has been made, and grasp one horn of the uterus and bring it with its contents through the incision to the outside, as it is impossible to prevent the escape of the liquor amnii and small particles of placenta into the abdominal cavity if the uterus is left in the abdomen while the contents are being removed. The incision into the uterus should be made, when practicable, between two pigs, so that they may both be removed through the same incision. More than two may be removed through one incision if it can be done without irritating the parts in trying to get the pigs to one opening. Rather than cause any unnecessary irritation of the uterus it is better to make an incision for each fœtus. After the fœtuses have been removed take all the membranes and fluids from the uterus; then close the incision in the uterus with an uninterrupted silk suture, so as to turn the edges in and bring the peritoneal coats together; then this horn should be replaced and the other horn grasped and brought to the outside in the same manner as the first. Should there be any fœtuses in the fundus of the uterus they should be gently pressed back into the horn of the uterus and removed in the same way as the others. The incision in the uterus should be closed by a continuous silk suture, care being taken to turn the edges in so as to bring the peritoneal surfaces together. The abdominal incision should be closed by passing a suture through the skin, muscle, and peritoneum. Little or no after-treatment is advisable. With this method of operating, in the summer of 1900 I had eight cases out of eleven to make speedy and successful recoveries. Three of these were operated upon for Mr. D. C. Rook, a Poland China breeder, of Oakley, by lantern light, and in twenty-five days were sold and shipped to Chicago. Last season out of ten cases seven made good recoveries. One was a case of extra-uterine pregnancy which made a good recovery and sold in a short time after the operation for about $25.

### Discussion.

In answer to a question Dr. Stewart said that he gives an anæsthetic. He uses chloroform.

Dr. Heck has performed a number of Cæsarean sections upon sows, with a mortality of only 15 per cent. He uses chloroform as an anæsthetic. He utilizes interrupted silk sutures. He disinfects his silk by soaking a few days in formaldehyde solution, and then carries them in absolute alcohol.

Dr. Walrod said that he has done a number of these operations, and that he always induces chloroform anæsthesia. His objection is that the operation does not command a fee commensurate with the difficulty of the operation.

Dr. Stewart said that in one case he secured a fee of five dollars; that he has never driven over a mile to do the operation, and that he operates partly because he has a scientific interest in such cases. This interest was gratified in one case by finding a case of extra-uterine pregnancy—the only case he had ever seen in a domestic animal.

Dr. D. H. Miller said that he uses an anæsthetic, as he fears death from shock without its use.

